# Nd^3+^-Doped Scheelite-Type Multifunctional Materials—Their Thermal Stability and Magnetic Properties

**DOI:** 10.3390/ma17194883

**Published:** 2024-10-04

**Authors:** Elżbieta Tomaszewicz, Grażyna Dąbrowska, Hubert Fuks, Paweł Kochmański

**Affiliations:** 1 Department of Inorganic and Analytical Chemistry, Faculty of Chemical Technology and Engineering, West Pomeranian University of Technology in Szczecin, Al. Piastów 42, 71-065 Szczecin, Poland; grazyna.dabowska@zut.edu.pl; 2 Faculty of Mechanical Engineering and Mechatronics, West Pomeranian University of Technology in Szczecin, Al. Piastów 19, 70-310 Szczecin, Poland; hubert.fuks@zut.edu.pl (H.F.); pawel.kochmanski@zut.edu.pl (P.K.)

**Keywords:** scheelites, Nd^3+^ ion, solid solution, thermal stability, magnetic properties

## Abstract

New Nd^3+^-doped cadmium molybdato-tungstates with the chemical formula of Cd_1−3*x*_▯_x_Nd_2*x*_(MoO_4_)_1−3*x*_(WO_4_)_3*x*_ (where *x* = 0.0283, 0.0455, 0.0839, 0.1430, 0.1875, 0.2000, 0.2500, and ▯ denotes a vacant site in the crystal lattice) were successfully synthesized by the high-temperature solid state reaction method, using CdMoO_4_ and Nd_2_(WO_4_)_3_ as the initial reactants. The structure and change in their lattice parameters as a function of Nd^3+^ ion concentration were investigated by the XRD (X-ray diffraction) method. The surface morphology and grain size of the doped materials were characterized by SEM (scanning electron microscopy). Their thermal properties and initial reactants were analyzed by DTA-TG (differential thermal analysis coupled with thermogravimetry) techniques. The optical properties of the Nd^3+^-doped cadmium molybdato-tungstates, such as optical band gap, were determined by UV–vis–NIR (ultraviolet–visible–near infrared) spectroscopy. The EPR (electron paramagnetic resonance) technique provided information on the type of magnetic interactions between Nd^3+^ ions.

## 1. Introduction

Among the family of rare earth ions, Nd^3+^ is the most popular due to the presence of intense emission in the visible and near infrared ranges as well as its easy excitation with commercial diode lasers. Neodymium ion-doped amorphous or crystalline materials have been extensively studied and applied in the field of high-power lasers and optical communication [1,2,3,4,5].

In the last few years, Nd^3+^ has also been one of the most widely studied lanthanide ions for bio-sensing and bio-imaging applications [6,7,8]. The reason for this is the presence of excitation and emission lines within the first biological transparency window in the NIR region, i.e., within 700–950 nm [6,7,8]. In this spectral range, light absorption and scattering by biological tissues are strongly reduced. This makes materials activated by Nd^3+^ ions promising contrast agents for in vivo and in vitro fluorescence imaging. Many of them are also multifunctional, so that they can be applied both as probes for non-invasive early detection of malignant tumors and as agents for therapeutic purposes (localized hyperthermia).

The NIR emission originating from the ^4^F_3/2_ → ^4^I_9/2_ transition of Nd^3+^ ion has proven to be useful in temperature and pressure sensors [9,10,11,12,13,14,15,16]. Nd^3+^-doped nanocrystals such as NaYF_4_:Nd^3+^ could be used as sensors for precise temperature monitoring inside biological environments [10]. However, the performance of Nd^3+^-based optical sensors is significantly dependent on the choice of host material. The optical properties of temperature sensors based on Nd^3+^-doped metallic oxides, garnets, fluorides, perovskites, phosphates, and tungstates have been studied by many researchers [9,10,11,12,13,14,15,16].

Our research group has been dealing with Nd^3+^-doped materials for many years [17,18,19,20,21,22,23,24,25,26,27,28,29]. We have successfully introduced Nd^3+^ ions into many hosts with various types of structures, such as the scheelite type (CaMoO_4_, CdMoO_4_, PbMoO_4_, and PbWO_4_; space group *I*4_1_/*a*) [18,21,22,23,26,27,28], related to the wolframite type, i.e., ZnY_4_W_3_O_16_ [17] and CdY_2_W_2_O_10_ [18]; fluorite type (Y_6_MoO_12_, space group *Fm*$\bar{3}$*m*) [24]; monoclinic La_2_Mo_2_O_9_ (space group *P*2_1_) [19]; cubic La_2_MoWO_9_ (space group *P*2_1_3) [25]; and eulytite (M_3_Y(PO_4_)_3_, space group *I*$\bar{4}$3*d*) [29]. These Nd^3+^-doped materials were obtained in the form of micro- and nanocrystalline powders, single crystals, and transparent or partially transparent ceramics [17,18,19,20,21,22,23,24,25,26,27,28,29].

Our previous research on a microcrystalline Cd_1−3*x*_▯_x_Nd_2*x*_MoO_4_ solid solution with the scheelite-type structure and different Nd^3+^ ion concentrations (where ▯ denotes vacant site) showed the disorder in the crystal lattice around Nd^3+^ ions resulting from the presence of cation vacancies. This phenomenon is manifested by an emission of broad bands associated with *f*-*f* transitions in Nd^3+^ [21]. This clearly observed broadening of emission lines allows for both the tuning of laser radiation within the range of 1030–1080 nm as well as the generation of ultra-short pulses that could find application in pico- or femto-second crystalline lasers [21].

Other materials such as commercial high-speed silicon (Si) or silicon nitride (Si_3_N_4_) have found applications in optoelectronics and photonics [30,31]. They have been successfully used for developing photonic integrated circuits applied in next-generation high-speed data transmission [30,31].

Among the many types of structures represented by metal tungstates and molybdates, the scheelite-type structure is noteworthy. Divalent metal molybdates with relatively large cations such as Ca^2+^, Sr^2+^, Ba^2+^, Cd^2+^, and Pb^2+^ (ionic radius above 0.99 Å) exist in the scheelite-type structure (tetragonal symmetry, space group *I*4_1_/a; Z = 4), where molybdenum ions adopt tetrahedral coordination, while divalent metal ions represent an eight-coordinated position [32]. Tetragonal scheelite-type crystals, both the un-doped and those activated with RE^3+^ ions, form a wide and important class of inorganic materials that have a high application potential in various fields such as scintillator detectors, phosphors, and electro-optic devices [33,34,35].

This work is a continuation of our research on new multifunctional optical and magnetic materials. Here, we synthesized a new solid solution based on Nd^3+^-doped cadmium molybdato-tungstates. We determined the homogeneity range of this solution, as well as its structural, thermal, and magnetic properties.

## 2. Materials and Methods

### 2.1. Synthesis of CNMWO Solid Solution

New doped materials, i.e., microcrystalline samples of solid solution with the chemical formula of Cd_1−3*x*_▯*_x_*Nd_2*x*_(MoO_4_)_1−3*x*_(WO_4_)_3*x*_, where *x* = 0.0283, 0.0455, 0.0839, 0.1430, 0.1875, 0.2000, and 0.2500, and ▯ represents vacancies in the crystal lattice (described hereafter as CNMWO), were obtained via two-step synthesis. High-temperature sintering of appropriate reactants was applied in both steps of the synthesis. In the first step of the synthesis, cadmium molybdate (CdMoO_4_) and neodymium tungstate (Nd_2_(WO_4_)_3_) were obtained according to our previous studies [21,22,27]. As the initial reactants, tungsten oxide (WO_3_), molybdenum oxide (MoO_3_), and cadmium oxide (CdO) were used. Only neodymium oxide (Nd_2_O_3_) was subjected to thermal pretreatment, i.e., it was heated at 900 °C for 12 h. In the second step of the synthesis, seven two-component mixtures containing Nd_2_(WO_4_)_3_ and CdMoO_4_, with the neodymium tungstate content varying from 3.00 to 50.00 mol%, were prepared. The composition of all prepared mixtures is provided in Table A1. The samples were sintered in corundum crucibles through several 12-h heating stages, at temperatures ranging from 900 °C to a maximum of 1075 °C. After each heating step, the samples were ground, and their weight was monitored. A slight mass loss (less than 0.10%) indicated that the solid-state reaction between CdMoO_4_ and Nd_2_(WO_4_)_3_ occurred with virtually no change in mass. This reaction can be described by the following general equation:

(1 − 3*x*) CdMoO_4_ + *x* Nd_2_(WO_4_)_3_ = Cd_1−3*x*_▯*_x_*Nd_2*x*_(MoO_4_)_1−3*x*_(WO_4_)_3*x*_ (1)

The formula of each obtained sample is shown in Table A1.

### 2.2. Characterization of Methods

X-ray diffraction analysis was performed on an EMPYREAN II diffractometer (PANalytical, Almelo, The Netherlands) using CuKα_1,2_ radiation (λ = 1.5418 Å). Powder diffraction patterns of all samples were recorded in the 10–80° 2 Θ range with a scanning step of 0.013°, and next analyzed by HighScore Plus 4.0 software. The lattice parameters were calculated using POWDER 2.0 software [36,37].

Simultaneous DTA-TG measurements were carried out using a TA Instruments thermoanalyzer (model SDT 2960, New Castle, DE, USA). The detailed conditions of these studies have been described in our previous works [19,21,27].

The grain size, morphology, and elemental composition of certain CNMWO materials were examined using a field emission scanning electron microscope (FE-SEM), model Hitachi SU-70 (Hitachi, Naka, Japan). The detailed conditions of these studies are described in references [21,24,25].

The optical properties in the UV–vis–NIR region were studied using a JASCO V-670 spectrophotometer (JASCO Europe S.R.L., Cremella, Italy). The detailed experimental conditions are described in references [22,25,27].

EPR spectra of Nd^3^^+^-doped materials were recorded on a conventional X-band Bruker ELEXSYS E 500 CW spectrometer (Bruker, Biellerica, MA, USA) operating at 9.5 GHz with 100 kHz magnetic field modulation. The first derivative of the absorption spectra was recorded as a function of the applied magnetic field. The temperature dependence of the EPR spectra of the solid solutions under study was measured in the 4–300 K range using an Oxford Instruments ESP helium-flow cryostat.

## 3. Results and Discussion

### 3.1. X-ray Diffraction Studies of CNMWO Materials

As mentioned in the Section 1, cadmium molybdate exhibits a tetragonal scheelite-type structure at room temperature. It is also known that this compound does not exhibit polymorphism [32].

**Figure 1:**
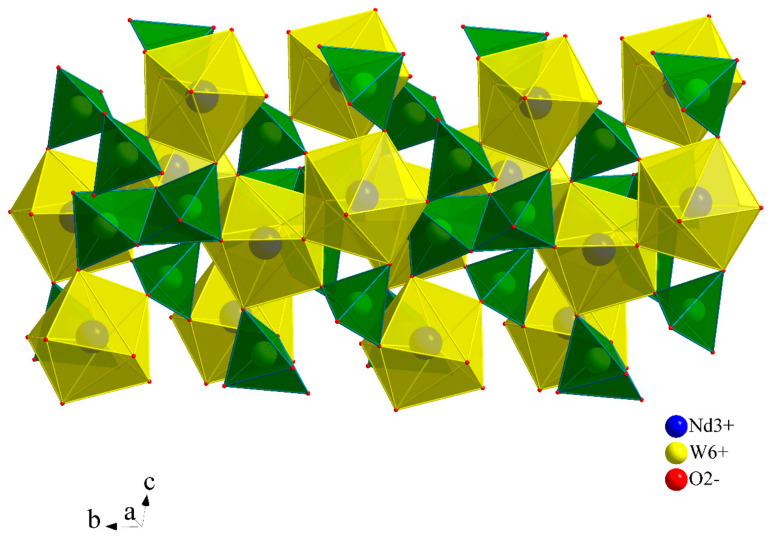
Crystal structure model of α-Nd_2_(WO_4_)_3_, where NdO_8_ polyhedra are in yellow; WO_4_ tetrahedra are in green.

The second initial reactant, Nd_2_(WO_4_)_3_, crystallizes at room temperature in a monoclinic symmetry (a = 7.7589(12) Å; b = 11.597(2) Å; c = 11.516(2) Å; β = 109.561°; Z = 4; space group C2/c) [38]. This polymorphic modification of neodymium tungstate (α-form) is a member of the Eu_2_(WO_4_)_3_ structural family and is isotypic with other RE^3^^+^ molybdates and tungstates of this formula type. Its structure can be derived from the scheelite-type structure, where in a threefold supercell, one Nd^3^^+^ crystallographic position is unoccupied. Neodymium ions are coordinated by eight O^2^^−^ ions in the form of a distorted bicapped trigonal prism. Two unique W^6^^+^ ions are tetrahedrally surrounded by O^2^^−^ ions, forming slightly and strongly deformed tetrahedra. Both polyhedra form W_2_O_8_ dimers through edge-sharing. The WO_4_ and NdO_8_ units combine to create a three-dimensional crystal lattice (Figure 1).

**Figure 2:**
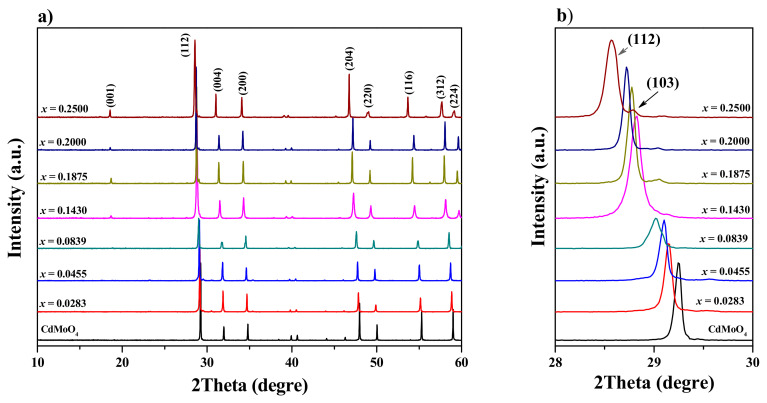
Powder XRD patterns of CdMoO_4_ and CNMWO samples when 0 < *x* ≤ 0.2500 in the 2Theta range of 10–60° (**a**); powder diffraction patterns in the 2Theta range from 28 to 30° with (*112*) and (*103*) diffraction lines (**b**).

**Figure 3:**
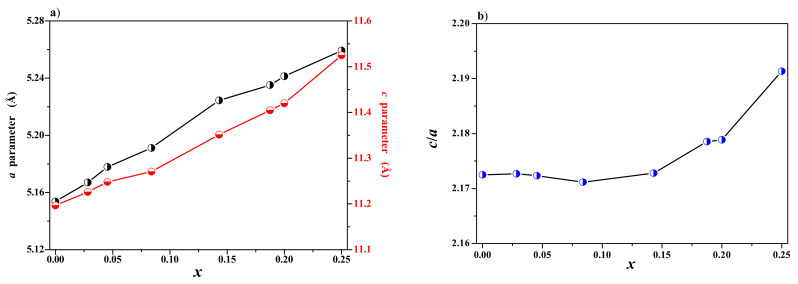
Variation in *a* and *c* unit cell parameters (**a**) and lattice parameter ratio *c*/*a* (**b**) of CNMWO materials as the function of *x* value.

Powder diffraction patterns of CdMoO_4_ and CNMWO solid solution samples with varying Nd^3^^+^ ion concentrations (*x* = 0.0283, 0.0455, 0.0839, 0.1430, 0.1875, 0.2000, and 0.2500) are shown in Figure 2a,b. The XRD results for the CNMWO materials revealed that these patterns consisted solely of diffraction lines corresponding to the scheelite-type framework. No impurity compounds, such as metal oxides (CdO, Nd_2_O_3_, MoO_3_, and WO_3_), or other neodymium tungstates or molybdates were detected in the CNMWO samples after thermal treatment. It was also observed that all peaks attributed to the scheelite-type structure shifted clearly towards lower 2θ angles with increasing Nd^3^^+^ ion content (Figure 2b). The change in the 2Θ position of the diffraction line with the highest intensity (*112*) is shown in Figure 2b. All observed peaks were successfully indexed to the pure tetragonal scheelite-type structure (space group I41/a, No. 88, CdMoO_4_—JCPDS No. 01-088-0182). The calculated lattice constants are presented in Table A1. Figure 3a,b show the variation in unit cell parameters (a and c) and the lattice parameter ratio (*c*/*a*) as a function of Nd^3^^+^ concentration. Both lattice parameters of the doped materials increase with the rising concentration of Nd^3^^+^ ions. This change is nearly linear, indicating that the unit cell parameters of the CNMWO samples follow Vegard’s law (Figure 3a). A noticeable expansion of the crystal lattice is observed, despite the fact that in the CdMoO_4_ structure, cadmium ions (Cd^2^^+^ CN = 8, ionic radius = 1.10 Å) are replaced by Nd^3^^+^ ions (Nd^3^^+^ CN = 8, ionic radius = 1.109 Å), which have a very similar radius [39]. We have previously observed a similar phenomenon in analogous vacancy solid solutions, namely, a significant expansion of the crystal lattice when large ions in certain matrices were replaced by much smaller ones [27,40]. For divalent metal molybdates and tungstates with a scheelite-type structure and the chemical formula M^II^Mo(W)O4 (M^II^ = Ca, Sr, Ba, Pb, and CdMoO4), Mo^6+^ and W^6+^ ions are tetrahedrally coordinated by oxygen ions, and their ionic radii are very similar, i.e., 0.41 Å and 0.42 Å, respectively [39]. Therefore, substituting Mo^6+^ ions in MoO_4_ with W^6+^ ions does not significantly change the *a* and *c* parameters. Similar to other vacancy solid solutions we have previously synthesized, the significant expansion of the crystal lattice observed in CNMWO materials is caused by point defects in their structure. Specifically, when three crystallographic positions of Cd^2^^+^ in CdMoO_4_ are substituted by only two Nd^3^^+^ ions, cation vacancies occur. The lattice parameter ratio (*c*/*a*) was also calculated (Figure 3b). This ratio is very close to that of the pure matrix, i.e., *c*/*a* = 2.1725, and remains stable for Nd^3^^+^ concentrations of *x* = 0.0283, 0.0455, 0.0839, and 0.1430. However, for other CNMWO samples, where *x* > 0.1430, the lattice parameter ratio increases noticeably. This indicates that, at higher Nd^3^^+^ concentrations, deformation of the tetragonal scheelite-type cell in CNMWO materials occurs.

### 3.2. Thermal Stability and Morphology of CNMWO Solid Solution

Knowledge of the thermal properties of new materials, such as melting point and the onset of thermal decomposition, is crucial for determining their potential applications. Simultaneous DTA-TG studies were conducted to investigate the thermal stability of the new Nd^3^^+^-doped materials. However, prior to these thermal studies, analogous measurements were performed on the initial reactants.

**Figure 4:**
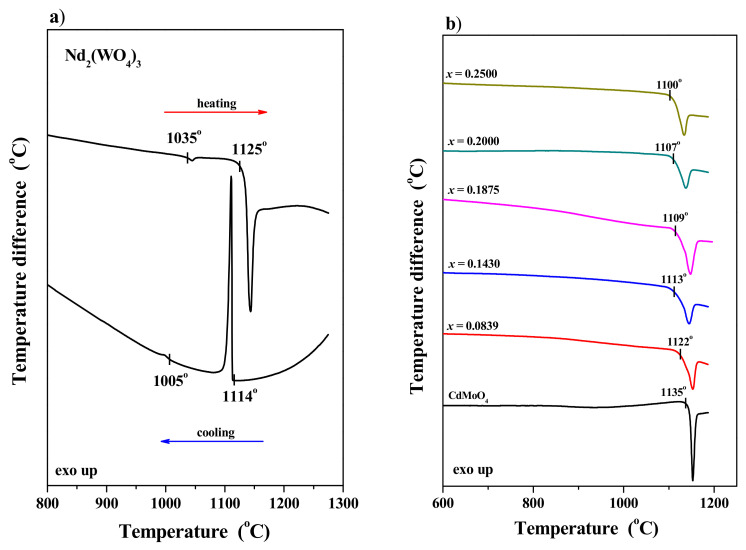
DTA curves of Nd_2_(WO_4_)_3_ recorded during controlled heating and next cooling this compound in the temperature range of 20–1270 °C (**a**); DTA curves of CdMoO_4_ and CNMWO materials recorded during heating them from 20 to 1200 °C (**b**).

The conducted studies showed that cadmium molybdate melts congruently at 1135 °C (Figure 4b), confirming the results of our earlier studies on this matrix [21,22]. There is relatively little information on the thermal properties of the second initial reactant, neodymium tungstate [41]. Figure 4a shows the DTA curves recorded during the heating and subsequent cooling of Nd_2_(WO_4_)_3_ (TG curves are not shown here). Two endothermic effects were observed in the DTA curve recorded during the controlled heating of neodymium tungstate. The first effect, starting at 1035 °C, is associated with the phase transformation of monoclinic α-Nd_2_(WO_4_)_3_ to a high-temperature polymorph (β) with orthorhombic symmetry (space group *Pnca*, Sc_2_(MoO_4_)_3_-type structure) [42,43]. A similar polymorphic transformation was observed for other rare earth tungstates, such as Pr_2_(WO_4_)_3_ [44] and Gd_2_(WO_4_)_3_ [45]. The second effect recorded on the DTA curve, beginning at 1125 °C, is related to the congruent melting of neodymium tungstate. The crystallization process from the melt and the phase transition from β- to α-polymorph start at temperatures of 1114 and 1005 °C, respectively (Figure 4a). Our DTA studies revealed that the polymorphic transformation of Nd_2_(WO_4_)_3_ is a reversible process. Figure 4b also shows the DTA curves of some CNMWO samples. Only one endothermic effect was observed on each DTA curve, attributed to the melting of each Nd^3+^-doped cadmium molybdate–tungstate. No mass losses were recorded on the TG curves (not presented here) up to the onset of the observed effects on the DTA curves. The results of the DTA studies indicate that as the content of Nd^3+^ ions in the CNMWO samples increased, the melting point of the samples decreased, from 1122 °C when *x* = 0.0839 to 1100 °C when *x* = 0.2500.

**Figure 5:**
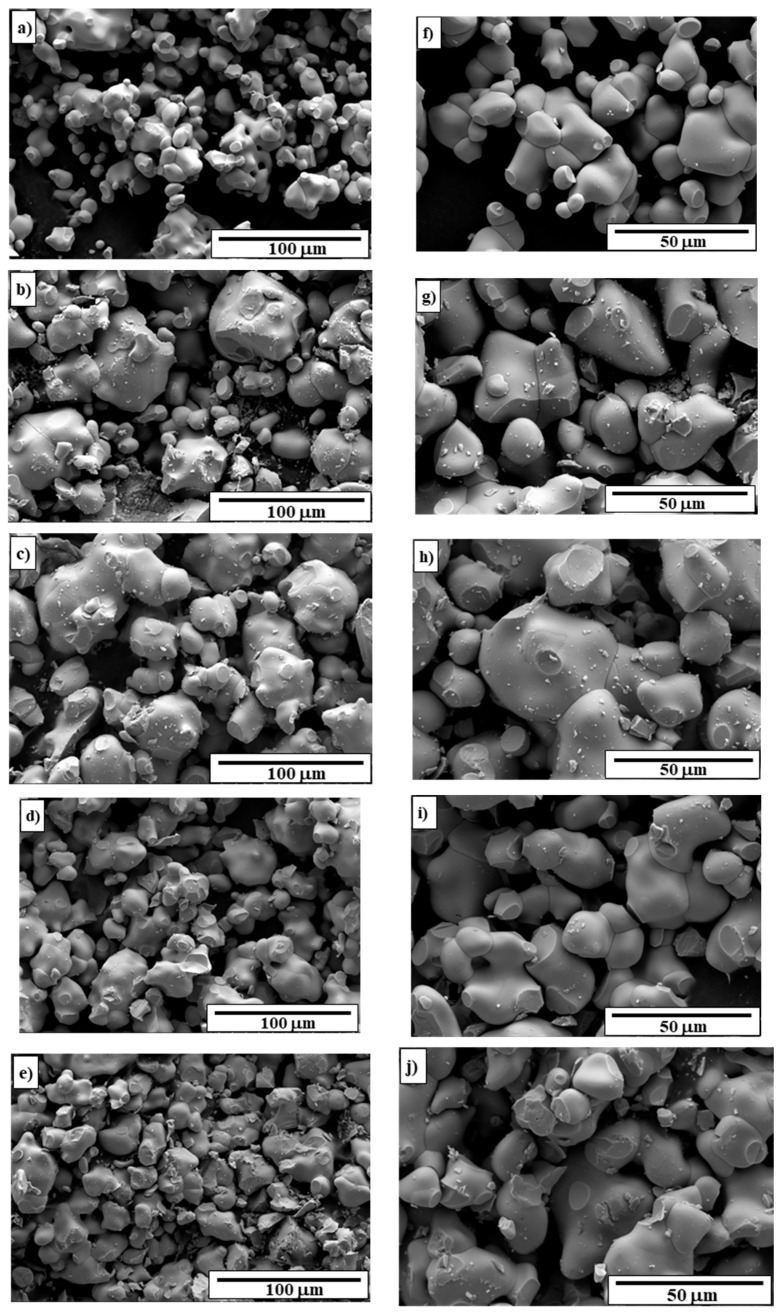
SEM images of CdMoO_4_ (**a**,**f**) and CNMWO samples when *x* = 0.0283 (**b**,**g**), *x* = 0.0839 (**c**,**h**), *x* = 0.1875 (**d**,**i**), and *x* = 0.2500 (**e**,**j**).

SEM micrographs of CdMoO_4_ and CNMWO samples with *x* values of 0.0283, 0.0839, 0.1875, and 0.2500 are shown in Figure 5a–j. Both cadmium molybdate and all Nd^3^^+^-doped samples consist of grains with well-defined and sharp boundaries, suggesting that the obtained micropowders are well-crystallized. The pure matrix contains small, uniform, oval grains with an average size not exceeding ~10 μm (Figure 5a,f). However, groups of connected particles with sizes approaching 30 μm can also be observed. The CNMWO samples exhibit a different morphology (Figure 5b–e,g–j). They contain non-uniform grains of varying sizes and shapes. Both the grain size and the number of large particles increase with the concentration of Nd^3^^+^ ion. The samples with a high amount of Nd^3^^+^ ion (*x* = 0.1875 and 0.2500) also contain agglomerates formed by the combination of individual grains. Large clusters composed of small particles exceed 50 μm in size.

EDX (energy dispersive X-ray) analysis revealed that the only elements present in the CNMWO materials were Cd, Nd, Mo, W, and O. These elements were evenly distributed on the surface of the analyzed samples, indicating that the obtained materials have a uniform chemical composition.

### 3.3. Optical Properties of CNMWO Materials

The optical properties of CdMoO_4_ and CNMWO microcrystalline samples were investigated at room temperature using UV–vis–NIR diffuse reflectance spectroscopy. This method enables the determination of important optical parameters for micro- and nanomaterials, as well as single crystals, including the absorption coefficient, band gap energy, and refractive index. Knowledge of these parameters is crucial for the future applications of these materials.

Cadmium molybdate, as one of the representatives of the family of compounds with a scheelite-type structure, exhibits a direct band gap. This means that valence electrons can be directly excited into the conduction band by a photon with energy greater than the band gap. Molybdates and tungstates, due to the absence of impurity levels between the valence band and conduction band, have high band gap values (E_g_) [46,47].

**Figure 6:**
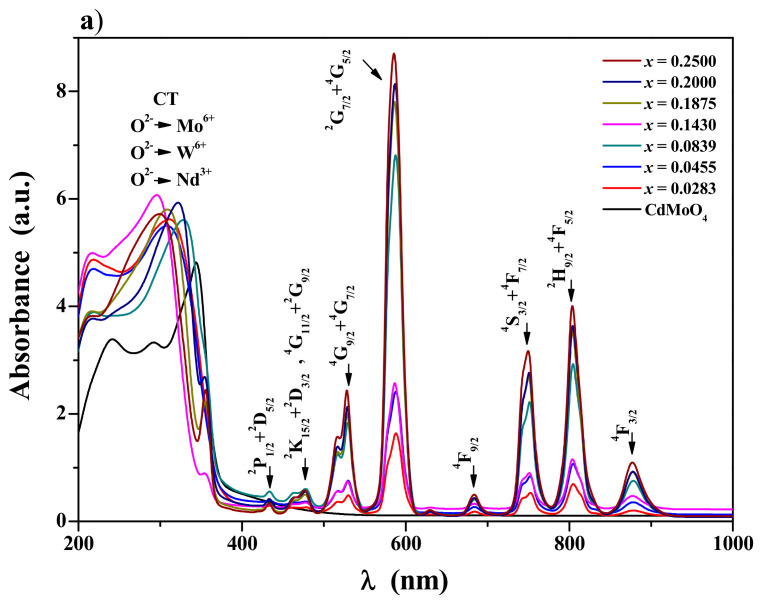

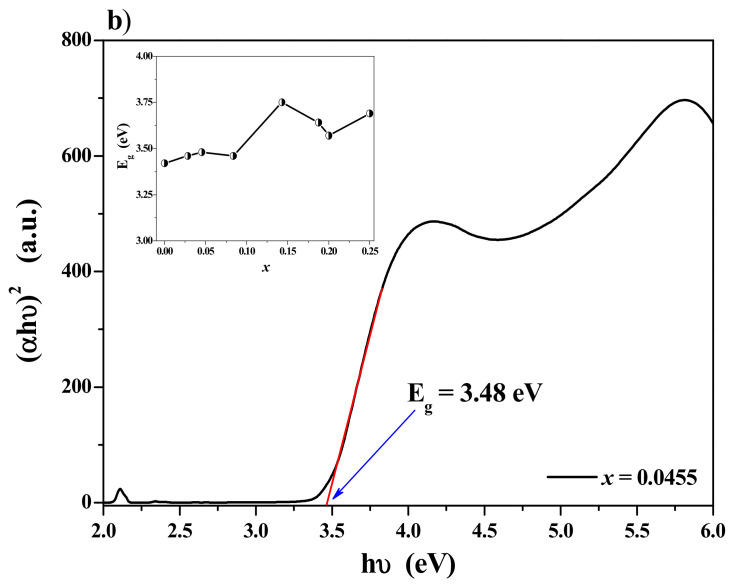
UV–vis–NIR absorption spectra of CdMoO_4_ and CNMWO samples with various values of *x* parameter (**a**); the plot of (*αhν*)^2^ vs. *hν* of CNMWO when *x* = 0.0455 as well as determined direct band gap energy (**b**): variation in E_g_ with value of *x* parameter (insert).

Figure 6a shows the UV–vis–NIR absorption spectra recorded at ambient temperature for CdMoO_4_ and CNMWO samples. All CNMWO materials exhibit energy absorption capabilities in the ultraviolet and visible ranges. The intense and broad absorption bands within the spectral range of 200–350 nm can be attributed to charge transfer bands involving O^2^^−^ → Mo^6^^+^, O^2^^−^ → W^6^^+^, and O^2^^−^ → Nd^3^^+^ [21,27]. Additionally, the intense but narrow bands observed at higher wavelengths in the visible region can be associated with transitions from the ground state ^4^I_9_/_2_ of the Nd^3^^+^ ion to its excited states (Figure 6a) [18,19,20,21,24,25,29]. In this work, we focused solely on the analysis of absorption spectra to determine the energy gap.

Optical band gap was determined using the Tauc relation [48,49].

(2) $$\alpha h\nu =A{\left(h\nu -E_{g}\right)}^{n}$$

where α is the absorption coefficient of the material under study, h is Planck’s constant, ν is the light frequency, A is a proportionality coefficient characteristic of the material, and n is a constant related to the nature of the electronic transition. For solid materials with a direct band gap, n = ½ [27,48,49]. The values of Eg were determined from the plot of (αhν)^2^ as a function of hν by extrapolating the linear portion of this relation to the photon energy axis (Table A1, Figure 6b) [27,46,47]. The optical band gap of the CNMWO samples increased as the Nd^3^^+^ ion content increased from x = 0.0283 to x = 0.1430 (Table A1, Figure 6b, insert). At the latter Nd^3^^+^ concentration, the highest E_g_ value was observed, i.e., 3.75 eV. However, with a further increase in Nd^3^^+^ concentration, the energy gap values decreased.

The systematic change in the optical band gap with Nd^3^^+^ doping is connected to structural disorder and on-site fluctuations, which arise due to the substitution of Nd^3^^+^ for Cd^2^^+^, W^6^^+^ for Mo^6^^+^ in the CdMoO_4_ framework, as well as the creation of vacancies according to the substitution: 3 Cd^2^^+^ → 2 Nd^3^^+^ + ▯. Additionally, there are significant differences in electronegativity between Cd (1.69) and Nd (1.14), as well as Mo (2.16) and W (2.36). These differences in electronegativity shift the valence band towards the conduction band, or vice versa, resulting in a decrease or increase in the band gap with doping.

### 3.4. EPR Studies of CNMWO Samples

**Figure 7:**
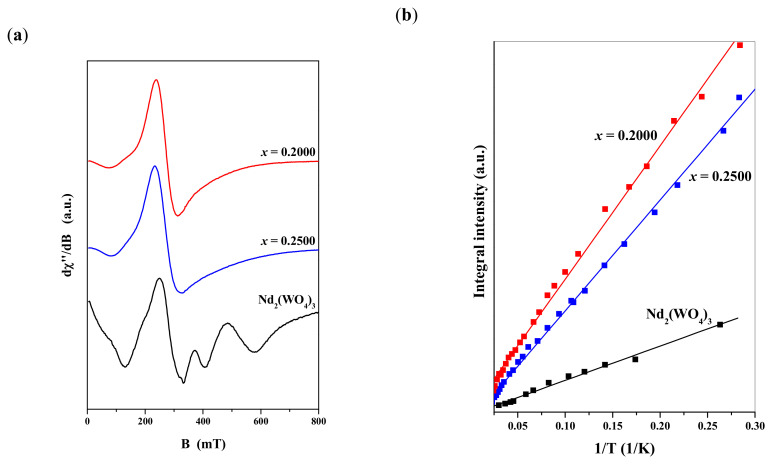
(**a**) Low-temperature EPR spectra of CNMWO with different Nd^3+^ ion concentration and spectrum of Nd_2_(WO_4_)_3_ sample. (**b**) Integral intensity of EPR signal as a function of reverse temperature. Lines represent results of simulation with employing Curie–Weiss law.

Additional information about the presented CNMWO arrangements can be obtained from EPR investigations. The X-band resonance spectra, observed from liquid helium temperatures up to room temperature, revealed the presence of a resonance signal detectable only below 35 K, attributed to trivalent neodymium ions. Figure 7a shows EPR spectra measured at approximately 7 K for two CNMWO samples with *x* = 0.2000 and *x* = 0.2500, along with the spectrum of Nd_2_(WO_4_)_3_, used as a reference sample.

For a well-isolated Nd^3^^+^ ion, the EPR signal typically consists of a single, narrow Lorentzian line, originating from an energy transition within the electronic spin S = ½. This resonance line can sometimes be influenced by hyperfine interactions with the nuclear magnetic moment (I = 7/2), leading to the appearance of weak satellite lines [50,51]. However, when the concentration of neodymium ions is higher, significant magnetic exchange interactions between Nd^3^^+^ ions occur and must be considered. As a result, the resonance signal becomes broader and more complex [52].

As could be seen in Figure 7a, the EPR spectra of CNMWO with *x* = 0.2000 and 0.2500 are close to a single resonance line, which differs significantly from the signal observed in the dense magnetic system of Nd_2_(WO_4_)_3_. This suggests that the magnetic arrangement in both CNMWO samples originates from relatively well-isolated magnetic Nd^3^^+^ ions. Some deviation from a pure Lorentzian shape could be due to the presence of vacancy states (▯_x_) in the materials, generated by the electronic compensation mechanism that occurs when some Cd^2^^+^ ions are replaced by Nd^3^^+^ ions in the crystal structure. These magnetically active vacancies interact with Nd^3^^+^ ions via magnetic exchange mechanisms, resulting in a resonance signal that deviates from the perfect line expected for well-isolated magnetic centers.

A similar change in the EPR signal was observed earlier in the scheelite-type structure Ca_1__−__3_*_x_*_−_*_y_*Mn*_y_*▯*_x_*Eu_2_*_x_*(MoO_4_)_1__−__3_*_x_*(WO_4_)_3_*_x_* [53], where the overall EPR spectra did not correspond to isolated Mn^2^^+^ or Eu^3^^+^ ions but were significantly broadened due to the mutual interaction between the two different magnetic centers.

For all samples, the EPR signal decreases with increasing temperature and disappears completely above 35 K. The integral intensity of the signal, I, follows the Curie–Weiss law: ***I*** = *C*/(*T* − *θ*) (Figure 7b), with a very low, near-zero temperature parameter *θ*. This confirms the generally paramagnetic behavior of the magnetic centers in both CNMWO samples. A similar result, with *θ* parameter close to zero, was observed in the dense magnetic system of Nd_2_(WO_4_)_3_.

In scheelite-type structures, the overall magnetic arrangement can be influenced by various factors, including the doping level of the magnetic ions, their uniform or non-uniform distribution, and their specific positions within the crystal structure. For example, in Cd_1__−__3_*_x_*▯*_x_*Gd_2_*_x_*MoO_4_, we observed ferromagnetic interactions among the gadolinium ions [54]. In contrast, in Pb_1__−__3_*_x_*▯*_x_*Gd_2_*_x_*(MoO_4_)_1__−__3_*_x_*(WO_4_)_3_*_x_*, the gadolinium ions exhibited strong antiferromagnetic (AFM) interactions [55]. A very interesting case was described in our work [56], where an unusual dependence of the EPR signal intensity on temperature was observed. We explained this behavior by the possible coexistence of ferromagnetic (FM) and antiferromagnetic (AFM) interactions between Gd^3^^+^ ions. This possibility was previously suggested by Skrzypek et al. [57], who proposed that FM interactions occur in a two-dimensional magnetic system, while AFM arrangements dominate in a three-dimensional magnetic system. Thus, we believe that the overall calculated *θ* value close to zero in our case results from a similar situation, namely, the combined interactions among the responsible Nd^3^^+^ and ▯*_x_* magnetic centers, with FM arrangements in the planes and AFM arrangements between neighboring planes containing the magnetically active elements.

## 4. Conclusions

In this study, a new Cd_1−3*x*_▯*_x_*Nd_2*x*_(MoO_4_)_1−3*x*_(WO_4_)_3*x*_ solid solution (*x* = 0.0283; 0.0455; 0.0839; 0.1430; 0.1875; 0.2000; 0.2500 and ▯ represents vacancy) was synthesized using the solid state reaction method. XRD analysis revealed that new Nd^3+^-doped materials crystallize in scheelite-type structure (tetragonal symmetry, space group *I*4_1_/*a*). The crystal lattice of all samples shows a strong expansion, and both lattice constants increase linearly with the increase in Nd^3+^ ion concentration. This phenomenon occurs despite the fact that in the CdMoO_4_ crystal lattice, the Cd^2+^ and Mo^6+^ ions are replaced by others with very similar radii. New Nd^3+^-doped cadmium molybdato-tungstates do not show polymorphism and melt at temperatures higher than 1100 °C. Information on the thermal stability of Nd^3+^-doped molybdato-tungstates is important from the point of view of their scope of application and the determination of pulling conditions of single crystals by the Czochralski method. Materials activated by Nd^3+^ ions show strong absorption in the ultraviolet and visible range. Intense absorption bands recorded in the range of 700–950 nm (the so-called first biological window) suggest their promising applications to the study of biological objects. All materials are electrical insulators, and their direct band gap nonlinearly changed with the increase in both doping ion concentrations.

In this study, a new solid solution of Cd_1__−__3__x_▯_x_Nd_2__x_(MoO_4_)_1__−__3__x_(WO_4_)_3__x_ (where *x* = 0.0283, 0.0455, 0.0839, 0.1430, 0.1875, 0.2000, 0.2500, and □ represents a vacancy) was synthesized using the solid-state reaction method. XRD analysis revealed that the new Nd^3^^+^-doped materials crystallize in a scheelite-type structure with tetragonal symmetry (space group *I41/a*). The crystal lattice of all samples shows significant expansion, and both lattice constants increase linearly with the increasing concentration of Nd^3^^+^ ions. This phenomenon occurs despite the fact that in the CdMoO_4_ crystal lattice, Cd^2^^+^ and Mo^6^^+^ ions are replaced by other ions with very similar radii. The new Nd^3^^+^-doped cadmium molybdato-tungstates do not exhibit polymorphism and melt at temperatures exceeding 1100 °C. Information on the thermal stability of Nd^3^^+^-doped molybdato-tungstates is crucial for their potential applications and for determining the conditions for growing single crystals using the Czochralski method. Materials activated by Nd^3^^+^ ions show strong absorption in the ultraviolet and visible ranges. Intense absorption bands recorded in the range of 700–950 nm (the so-called first biological window) suggest promising applications for the study of biological objects. All materials are electrical insulators, and their direct band gap changes nonlinearly with an increase in the concentrations of both doping ions.

EPR investigations of CNMWO phases confirm the existence of neodymium magnetic centers located within the Cd octahedral crystallographic positions. The vacancies (▯_x_) generated in this manner affect the magnetic arrangement of Nd^3^^+^ ions.

## Data Availability

The original contributions presented in the study are included in the article; further inquiries can be directed to the corresponding author.

## Appendix A

**Table A1:** Composition of initial Nd_2_(WO_4_)_3_/CdMoO_4_ mixtures, formula of Cd_1−3*x*_▯*_x_*Nd_2*x*_(MoO_4_)_1−3*x*_(WO_4_)_3*x*_ (CNMWO) solid solution, calculated *a* and *c* lattice parameters, *c*/*a* ratio, and determined direct band gap energy (E_g_).

Nd_2_(WO_4_)_3_ Content [mol%]	CdMoO_4_ Content [mol%]	Formula of CNMWO Solid Solution, Values of *x* Parameter		Lattice Parameters			E_g_ [eV]
				*a* [Å]	*c* [Å]	*c/a*	
0	100	*x* = 0	CdMoO_4_	5.15373(8)	11.1965(4)	2.1725	3.32
3.00	97.00	*x* = 0.0283	Cd_0.9151_▯_0.0283_Nd_0.0566_(MoO_4_)_0.9151_(WO_4_)_0.0849_	5.16702(9)	11.2263(6)	2.1727	3.46
5.00	95.00	*x* = 0.0455	Cd_0.8635_▯_0.0455_Nd_0.0910_(MoO_4_)_0.8635_(WO_4_)_0.1365_	5.17786(10)	11.2480(7)	2.1723	3.48
10.00	90.00	*x* = 0.0839	Cd_0.7483_▯_0.0839_Nd_0.1678_(MoO_4_)_0.7483_(WO_4_)_0.2517_	5.19115(8)	11.2708(7)	2.1712	3.46
20.00	80.00	*x* = 0.1430	Cd_0.5710_▯_0.1430_Nd_0.2860_(MoO_4_)_0.5710_(WO_4_)_0.4290_	5.22441(9)	11.3516(8)	2.1728	3.75
30.00	70.00	*x* = 0.1875	Cd_0.4375_▯_0.1875_Nd_0.3750_(MoO_4_)_0.4375_(WO_4_)_0.5625_	5.23509(11)	11.4048(5)	2.1785	3.64
33.33	66.67	*x* = 0.2000	Cd_0.4000_▯_0.2000_Nd_0.4000_(MoO_4_)_0.4000_(WO_4_)_0.6000_	5.24128(8)	11.4201(9)	2.1789	3.57
50.00	50.00	*x* = 0.2500	Cd_0.2500_▯_0.2500_Nd_0.5000_(MoO_4_)_0.2500_(WO_4_)_0.7500_	5.25931(12)	11.5249(6)	2.1913	3.69
